# Distribution, Diversity, and Ecological Risks of Microplastics in Mangrove Ecosystems of a Southeastern Chinese Estuary

**DOI:** 10.3390/toxics13060494

**Published:** 2025-06-12

**Authors:** Fengrun Wu, Chengyi Zhang, Xueyan Li, Sha Liu, Jinpu Wang, Weiqi Huang

**Affiliations:** School of Environmental Science and Engineering, Xiamen University of Technology, Xiamen 361024, China; 2322151036@stu.xmut.edu.cn (C.Z.); 15235771532@163.com (X.L.); 2422151015@stu.xmut.edu.cn (S.L.); 19991261453@163.com (J.W.); 17689906288@163.com (W.H.)

**Keywords:** microplastic, mangroves, sediment, estuary, ecological risk

## Abstract

Mangrove ecosystems, serving as critical barriers at land–sea interfaces, face increasing threats from microplastic pollution. This study investigates the spatial distribution, diversity, and ecological risks of microplastics in sediments from the Zhangjiang Estuary mangroves, in southeastern China. Sampling was conducted along two gradients: upstream–downstream and interior–edge habitats. The results revealed an average microplastic abundance of 219.5 ± 21.7 items·kg^−1^, dominated by fragments (53.3%) and fibers (35.0%). Additionally, 27.8% of the particles were in the 63–200 μm range, while 38.3% fell within the 200–500 μm range. A longitudinal decline in abundance from upstream to downstream was observed. Meanwhile, interior habitats exhibited significantly higher microplastic accumulation (292.86 ± 31.49 items·kg^−1^) than edge zones (142.50 ± 17.87 items·kg^−1^) (*p* < 0.05). The diversity index decreased downstream, with higher diversity in interior habitats, likely due to reduced terrestrial microplastic inputs and stronger tidal sorting in those areas. The ecological risk assessments indicated lower risks in Zhangjiang mangroves compared to global counterparts, though risks were elevated in interior habitats due to higher abundances of hazardous polymers (PVC, PS, PE). This study highlights the role of mangroves as microplastic sinks and advocates for multidimensional risk assessments integrating physical characteristics to guide conservation strategies in vulnerable estuarine ecosystems.

## 1. Introduction

Microplastics, defined as plastic particles ranging from 1 to 500 μm, are categorized into two types: primary microplastics, which are deliberately produced (e.g., microbeads in personal care products), and secondary microplastics, which result from the breakdown of larger plastics due to ultraviolet radiation, mechanical wear, or biological processes [1]. Global estimates suggest that around 8 million metric tons of plastic waste enter marine environments each year, with 15–31% eventually fragmenting into microplastics [2]. These particles are widely distributed, even in remote areas, such as mountain lakes [3], polar ice [4], and the Mariana Trench [5]. Microplastics serve as carriers for persistent organic pollutants (e.g., polycyclic aromatic hydrocarbons) and heavy metals, which accumulate in the food chain, leading to reproductive impairments, immune suppression, and behavioral changes in marine organisms [6,7]. Alarmingly, recent studies have detected microplastics in human blood [8] and placental tissues [9], with evidence linking chronic exposure to oxidative stress, inflammation, and cellular damage [10,11]. Unlike conventional pollutants that are often confined by geographical or temporal boundaries, microplastics represent a ubiquitous anthropogenic stressor that poses a significant threat to global ecological security [12].

Mangroves, located where rivers meet the sea, are vital ecosystems that protect coastlines and support diverse wildlife [13]. However, their dense root networks significantly reducethe intensity of hydrodynamics, enhancing the settling and trapping of fine-grained sediments and co-transported microplastics within the vegetation [14]. The asymmetric tidal forcing further promotes microplastic retention by limiting resuspension and export, favoring net accumulation over flushing to coastal waters [15]. For instance, in the Pearl River Estuary, mangrove areas were found to contain three to five times more microplastics (ranging from 560 to 2800 pieces per kg of sediment) compared to nearby bare mudflats. While most studies focus on comparing mangroves to surrounding areas, they often overlook the variations within the mangrove itself. Factors, such as water flow, salinity, and tree roots, likely influence the distribution of microplastics within the mangrove ecosystem [15,16]. To gain a more comprehensive understanding, it is essential to conduct a detailed sampling across different zones within the mangrove.

Emerging evidence underscores the necessity of multi-dimensional characterization in assessing mangrove microplastic pollution, extending beyond mere abundance quantification. The physical attributes (size, coloration, morphology) and polymer composition of microplastics serve as critical source identifiers and risk predictors. Fibrous morphologies typically signal urban wastewater inputs, whereas fragmented forms often indicate marine plastic degradation [17,18]. Chromatic characteristics further distinguish origins: black particles frequently associate with tire wear, while transparent films suggest packaging residues [19,20]. Polymer-specific risks manifest through distinct mechanisms: PVC-dominated particulates, constituting a major environmental burden, pose elevated ecotoxicity risks through chlorine leaching and disinfection byproduct formation during chlorination processes [21], whereas the extensive surface area of PET fibers enhances heavy metal adsorption capacity [22]. This dual analytical framework, combining spatial heterogeneity mapping through a multivariate characterization with trait-mediated ecological risk assessment, establishes critical decision-making foundations for mangrove conservation [23,24].

In this study, we investigate microplastic pollution in the sediment of the mangrove ecosystem at the Zhangjiang Estuary in southeastern China. By establishing two directional gradients—one from upstream to downstream and the other from the interior to the edge of the mangrove—we aimed to address three key questions: first, what is the spatial distribution pattern of microplastics in the mangrove sediments at the Zhangjiang Estuary? Second, how does the diversity of microplastic characteristics change along these gradients? Third, how do the ecological risks associated with microplastics vary across these two gradients?

## 2. Materials and Methods

### 2.1. Study Area and Sampling Sites

Our study was conducted in the Zhangjiang Estuary, located at 117°23′ E, 23°55′ N, in Yunxiao County, Zhangzhou City, Fujian Province. The estuary covers an area of approximately 23.69 km^2^ and serves as the confluence of the Zhangjiang River and its tributaries. The river flows into the estuary, connecting to Dongshan Bay before eventually reaching the Taiwan Strait. This region has a subtropical monsoon climate, with a mean annual air temperature of 21.2 °C and an average annual precipitation of 1714.5 mm, most of which occurs during the summer months. The mean annual salinity of the tidal waters in the estuary is 10.5%. The mangrove zone in the Zhangjiang Estuary is approximately 50–100 m wide and is home to several dominant species, including *Sonneratia caseolaris*, *Bruguiera gymnorhiza*, *Kandelia candel*, and mixed stands of these species.

**Figure 1:**
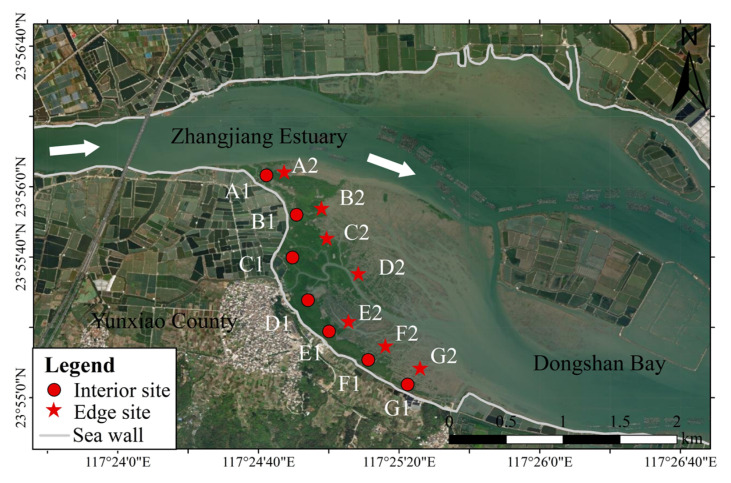
Geographical locations of the study sites in Zhangjiang Estuary, China. The base map is derived from Google Earth imagery, https://earth.google.com, accessed on 1 August 2023.

To investigate the distribution pattern of microplastics in the mangroves, we established seven sampling sites along a gradient from upstream to downstream in December 2023 (Figure 1). At each site, we sampled two distinct habitats: one inside the mangrove forest (interior habitat: approximately 30 m from the seawall) and one at the mangrove edge (edge habitat: less than 5 m from the unvegetated tidal flat). During low tide, sediment samples were collected using a stainless steel sediment corer with a 4.5 cm internal diameter. Three replicate sediment cores were taken at each habitat, with a sampling depth of 10 cm, and the samples were immediately stored in sealed aluminum containers. According to the study by Li et al. (2018) [25], the sedimentation rate at the Zhangjiang Estuary site was determined to be ~2.4 cm/year. Based on this rate, a sediment depth of 10 cm would represent approximately 4.2 years of accumulation. Given that the sampling was conducted in December 2023, the 10 cm sediment layer would correspond to materials deposited since 2019.

### 2.2. Laboratory Procedure

Microplastics were extracted from the sediment using the density separation method [26]. After thorough mixing, 20 g (±0.01 g) was taken from each sample and placed in a 500 mL beaker. To degrade the organic matter, 20 mL of 30% H_2_O_2_ (Sinopharm, Shanghai, China) was added and allowed to react for 24 h. A sodium chloride (NaCl) solution (1.12 g·L^−1^) was selected for density-based flotation due to its cost-effectiveness and broad applicability, with 300 mL aliquots added to each beaker. Notably, while this economical approach offers practical advantages, the methodology presents inherent limitations: the solution’s relatively low density compromises the recovery of high-density polymers. The sample was thoroughly stirred and then incubated in a 60 °C water bath for 36 h to allow the polymers to suspend in the supernatant. This process was repeated three times. Finally, the supernatant was filtered using a vacuum filtration system with 47 mm glass fiber filters (0.45 µm pore size).

Microplastics were examined under a microscope (MHAGO SZM0745T, Wuxi, China, 20 × 10 magnification) to observe their shape, color, and size (maximum dimension), and were recorded accordingly. The classification of microplastic morphology and color followed the methodology of Li et al. [27]. Particles smaller than 20 μm were excluded due to the FTIR detection limit; notably, the smallest particle size recorded in this study was 63 μm. For FTIR analysis, visually identified particles were transferred from glass fiber filters to Anopore membranes (Whatman™, Maidstone, UK, 0.2 μm) to minimize spectral interference, and analyzed using a Micro Fourier Transform Infrared Spectrometer (Thermo Fisher Scientific Nicolet iN10 MX, Waltham, USA) to identify polymer types. The spectral range was 4000 to 675 cm^−1^, with a resolution of 4 cm^−1^, a measurement time of 3 s per scan, and 16 scans were performed [28]. The spectra were processed using OMNIC™ Picta™ software (Version 1.5) and compared with reference databases to determine the polymer types [29]. Matches with a quality index ≥ 0.7 were accepted. 

### 2.3. Quality Assessment and Control

To minimize contamination, all researchers wore cotton lab coats and gloves, avoiding plastic products. All chemicals used in the experiments were filtered prior to use. All reagent additions, filtration steps, and sample handling were performed in a fume hood. Laboratory equipment, including aluminum containers, sediment corers, beakers, and Petri dishes, were thoroughly cleaned and rinsed twice with filtered pure water. During the procedures, all open containers were covered with aluminum foil to prevent contamination from airborne microplastics. For static incubation steps, containers remained covered with aluminum foil outside the fume hood. Three procedural blank controls were conducted to check for potential contamination, which were examined by visual microscopy, and no particles in the instrumentation size range capability were found in any of them.

### 2.4. Data Analysis

Data was processed using IBM SPSS Statistics 21 software. To assess the significance of variations in microplastic abundance and size across different sites and habitats, we conducted the Shapiro–Wilk test (two-tailed) to check the normality of the datasets. If the data deviated from normality, a square root transformation was applied to meet the normality requirement. In cases where normality could not be achieved, the Kruskal–Wallis test was performed, followed by pairwise comparisons. A significance level of 0.05 (*p*-value) was applied to all statistical analyses.

Additionally, the diversity of microplastics at different sites and habitats was assessed using the Shannon-Wiener index (*H*′), Pielou’s index (*J*′), and Simpson index (*D*). Microplastics collected from all sites were first categorized based on three criteria: color, shape, size, and polymer type. The Shannon–Wiener index quantifies the uncertainty in the composition of assemblages, while Pielou’s index measures the evenness of the communities. The Simpson index evaluates species dominance and diversity [27]. The indices were calculated as follows:

(1) $$H^{\prime }=-\sum_{i=1}^{S}P_{i}\ln P_{i},$$

(2) $$J^{\prime }=\frac{H^{\prime }}{\ln S}$$

(3) $$D=1-\sum P_{i}\times P_{i}$$

where *S* is the number of categories and *P_i_* is the proportion of categories *i* [30].

The pollution load index (PLI), polymer hazard index (PHI), and potential ecological risk index (PERI) were employed to evaluate the ecological risks of microplastic pollution in this study. The PLI quantifies the degree of microplastic pollution, the PHI assesses the risk posed by different polymer types, and the PERI determines the overall ecological risk [31]. Given by:

(4) $$PLI=\sqrt{\frac{C_{i}}{C_{0i}}}$$

(5) $$PHI=\sum (S_{n}\times P_{n})$$

(6) $$T_{r}^{i}=\sum_{n=1}^{n}\frac{P_{n}}{C_{i}}\times S_{n}$$

(7) $$C_{f}=\frac{C_{i}}{C_{0i}}$$

(8) $$PERI=\sum (T_{r}^{i}\times C_{f})$$

where the subscript *i* refers to the sample location. *C_i_* is the abundance of microplastics at the sampling site; *C*_0_*_i_* is the lowest observed MP concentration from all sites, used as the baseline value, following the method described by Yang et al. [32]; *S_n_* is the hazard score of each polymer type [33]; *P_n_* is the proportion of each polymer type in the sample; *T_r_* is the toxicity coefficient for the polymer; and *C_f_* is the contamination factor. The hazard scores of microplastic polymer types are provided in Table S1. The criteria for the risk levels based on the risk index and pollution load index are shown in Table S2.

## 3. Results

**Figure 2:**
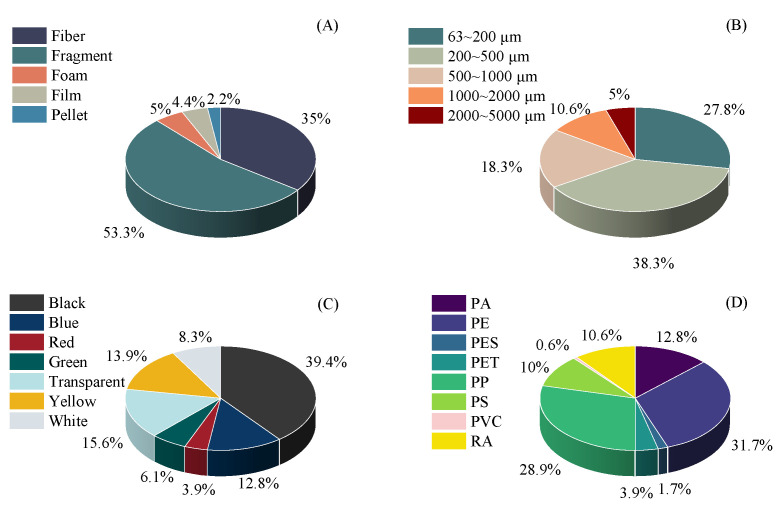
Shape, size, color, and polymer type composition of the microplastics in the mangroves at Zhangjiang Estuary. Note: (**A**) shape, (**B**) size, (**C**) color, and (**D**) polymer type composition of microplastics. PA, Polyamide; PE, Polyethylene; PES, Polyester; PET, Polyethylene terephthalate; PP, Polypropylene; PS, Polystyrene; PVC, Polyvinyl chloride; RA, Rayon. Data for the pie charts were derived from the integrated dataset combining all sampling points across all sites.

A total of 180 microplastic particles were detected in all sediment samples combined, with an average abundance of 219.5 ± 21.7 items·kg^−1^ (mean ± se). Among 42 sediment samples, we identified microplastic particles exhibiting five shape categories: fibers, fragments, foams, films, and pellets. Fragment-shaped particles accounted for the highest proportion (53.3%), followed by fiber (35.0%) (Figure 2). Microplastic particles averaged 577.57 ± 51.73 μm in size, with 27.8% falling into the 63–200 μm range and 38.3% in the 200–500 μm range. Particles larger than 2000 μm showed low abundance (5.0%). Seven color types were identified, where black (39.4%) and transparent (15.6%) particles showed the highest occurrence. Polypropylene (PP) and polyethylene (PE) were identified as the predominant polymer types among microplastics in mangrove sediments of Zhangjiang Estuary, comprising 44% and 37% of total polymers, respectively.

**Figure 3:**
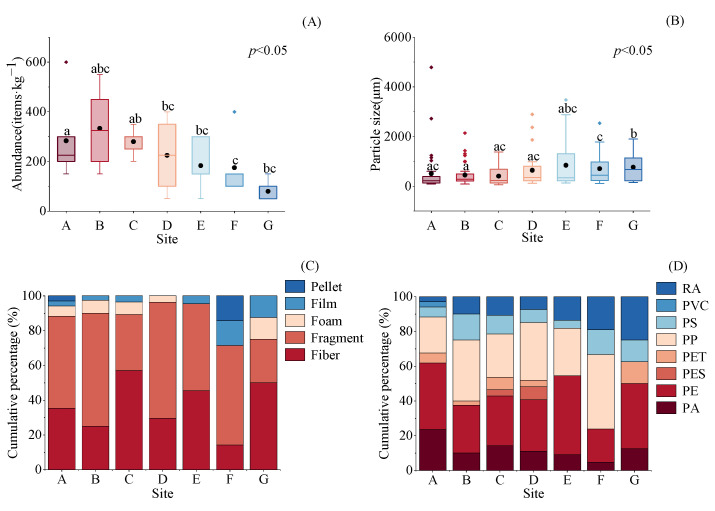
Longitudinal variations in the abundance, size, shape, and polymer type of microplastics from the upstream to downstream sites of the Zhangjiang Estuary. Note: (**A**) shape, (**B**) particle size, (**C**) color, and (**D**) polymer types of microplastics. The lines within boxes indicate medians, the black dots within boxes indicate means, the boxes indicate interquartile intervals, the triangles indicate outliers, and the bars above and below the boxes indicate the upper and lower non-outlier intervals. Different letters represent significant differences among groups at the 95% confidence level. Data from replicate sampling points at each site (e.g., A1 and A2, B1 and B2) were integrated to produce a merged dataset for each location.

There were significant spatial differences in the abundance of microplastics among different sites. The highest abundance was found at site B (333.3 ± 64.1 items·kg^−1^). Except for site B, the average abundance of microplastics showed a decreasing trend from the upstream to the downstream (Figure 3A). The average microplastic size varied significantly across the sites. Although there was no clear trend in size fluctuations, Site E exhibited the largest average microplastic size (846.84 ± 214.73 μm) (Figure 3B). The shape composition showed that fibers and fragments were the main types of microplastics at each site. However, the proportion of film and pellet microplastics increased at the downstream site F. In contrast, the proportion of foam and film microplastics was relatively high at site G (Figure 3C). PA, PE, PS, and RA were identified at all sites, with PP and PE being the dominant polymers, except at Site G, where PP was absent. Furthermore, the proportion of RA increased gradually from upstream to downstream along the river (Figure 3D).

**Figure 4:**
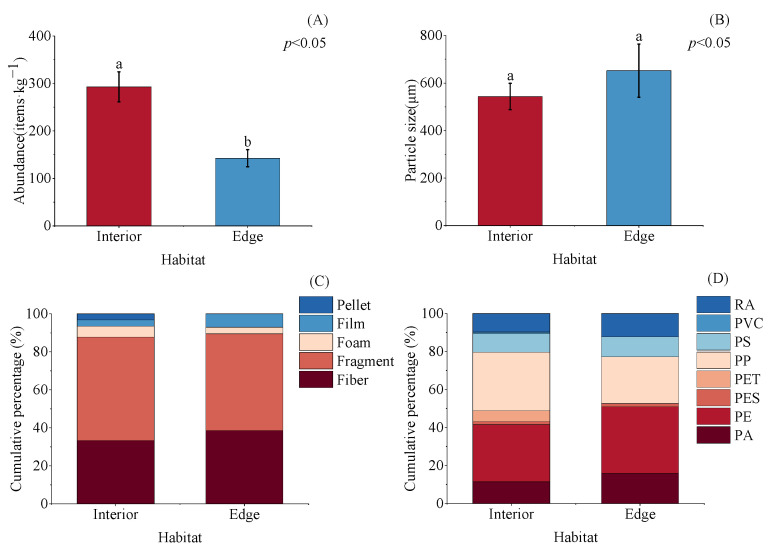
Variations in microplastic abundance, particle size, shape and polymers between habitats. Note: (**A**) shape, (**B**) particle size, (**C**) color, and (**D**) polymer types of microplastics. Different letters represent significant differences among groups at the 95% confidence level. Data from all interior sampling points (A1–G1) and all edge sampling points (A2–G2) were separately integrated to generate merged datasets for interior and edge habitats.

Compared to the edge habitat (142.50 ± 17.87 items·kg^−1^), microplastic abundance was significantly higher in interior habitats (292.86 ± 31.49 items·kg^−1^) (*p* < 0.05; Figure 4A). In contrast, although the average particle size in the interior habitat (543.08 ± 55.36 μm) was smaller than in the edge habitat (652.00 ± 111.63 μm), this difference was not statistically significant (*p* > 0.05; Figure 4B). Fragments and fibers remained the dominant shapes in both interior and edge habitats. However, notable spatial variations were observed: film particles were more prevalent in edge areas, while foam and pellet types showed a corresponding decrease (Figure 4C). Distinct differences in polymer type composition were also observed between habitats, with an increase in PE and a decrease in PP at the edge habitat. Additionally, PA was more abundant in edge sediments, whereas PET concentrations were higher in mangrove interiors (Figure 4D).

**Figure 5:**
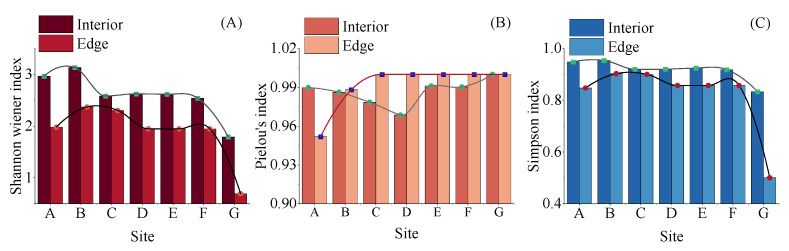
Variations in the diversity index of microplastics across different sites of the Zhangjiang Estuary. Note: (**A**) Shannon–Wiener’s index, (**B**) Pielou’s index, and (**C**) Simpson’s index.

The diversity of microplastics exhibited distinct patterns across habitats and sites. The Shannon–Wiener index in interior mangroves showed a longitudinal decreasing gradient across sites, consistently yielding higher values than in edge habitats (Figure 5A). Pielou’s index indicated elevated values in both interior mangrove and edge habitats, with fluctuations observed across sites, peaking at Site G (Figure 5B). The Simpson’s diversity index consistently showed higher values in interior mangroves compared to edge habitats across all seven sampling sites, with the lowest diversity observed in the edge habitat at Site G (Figure 5C).

The pollution load index (PLI) was low across all sites, with the highest value observed at Site B (2.58) (Table 1). This value exhibited a decreasing trend from upstream to downstream. The polymer hazard index (PHI) showed that Site A had a high polymer risk level, while the remaining sites were classified at a moderate level, with Site D showing the lowest PHI value (12.95). Additionally, the potential ecological risk index (PERI) for microplastics in sediments was at a low level at all sites; however, this value was higher at the upstream Site A compared to the other sites.

Both interior and edge results showed a low level of pollution loads, with the interior having a higher value. The polymer hazard index (PHI) showed differences between the interior, which had a high polymer risk level (506.18), and the edge, which had a moderate level (54.08). Although the potential ecological risk for microplastics in sediments was at a low level at all sites, the index was higher in the interior than in the edge (Table 2).

## 4. Discussion

As critical ecological barriers at land–sea interfaces, estuarine mangroves frequently experience intense anthropogenic pressures from activities such as fisheries and tourism. Understanding the occurrence characteristics of microplastics in their sediments is crucial for assessing coastal pollution and ecological risks. This study revealed an average microplastic abundance of 219.5 ± 21.7 items·kg^−1^ in Zhangjiang Estuary mangrove sediments, representing a low-to-moderate pollution level compared to global mangrove ecosystems. Notably, this value is substantially lower than those reported in other regions: Shenzhen Futian mangroves, China (2835 ± 713 items·kg^−1^) [34], Pearl River Estuary mangroves, China (851 ± 177 items·kg^−1^) [35], Bandar Abbas mangroves, Iran (3252 ± 2766 items·kg^−1^) [36], and Peruvian mangroves (726 ± 396 items·kg^−1^) [37]. This relatively low contamination levels may be attributed to effective conservation measures in the Zhangjiang Estuary Nature Reserve. The reserve strictly limits human access to core mangrove habitats, enforces bans on commercial fishing and aquaculture, and prioritizes mangrove restoration. Enhanced regulations on coastal development and waste management further reduce potential plastic inputs.

Morphological analysis showed dominant fragments (53.3%) and fibers (35.0%), aligning with patterns observed in China’s northern Beibu Gulf (50.4% fragments, 33.0% fibers) and Indonesian mangroves (52.2% fragments, 44.5% fibers) [38,39]. This consistency suggests terrestrial plastic waste (e.g., packaging materials and fishing nets) as the primary source of pollution. The high proportions of black (39.4%) and transparent (15.6%) microplastics further support this hypothesis: black particles likely originate from tire wear or industrial plastic debris, while transparent fragments may derive from plastic bags and films [40,41]. Notably, 66.1% of detected microplastics fell within the 63–500 μm size range, significantly exceeding proportions in other estuarine systems (e.g., 36.42% in Indonesian estuaries), potentially due to enhanced adsorption of fine particles by clay-rich sediments in Zhangjiang mangroves [42].

A longitudinal gradient of microplastic pollution, from upstream to downstream, is commonly observed in estuaries [43,44]. While previous studies have primarily focused on river channels and their sediments, research on this pattern in mangrove-fringed estuarine zones remains limited. In this study, the abundance of microplastics showed a significant decreasing trend from upstream to downstream (Figure 3A), with the exception of Site B, where elevated levels (333.33 ± 64.12 items·kg^−1^) were recorded. Field observations revealed a small fishing dock near this site, which likely contributed to the localized pollution peak. Similar spatial patterns of decreasing microplastic abundance from upstream to downstream have also been documented by Jiao et al. [45]. Two potential mechanisms may explain this distribution: first, upstream areas are often closer to terrestrial pollution sources, such as urban sewage and agricultural runoff, while enhanced tidal flushing downstream helps transport particles to open seas [46]. Notably, Pan et al. (2023) reported microplastic abundances of 55–184 items·kg^−1^ in sediments from Dongshan Bay, adjacent to the Zhangjiang Estuary, suggesting that downstream mangrove areas may act as secondary pollution sources [47]. Second, the cumulative hydrodynamic retardation caused by mangrove roots along the estuarine gradient effectively traps microplastics originating from upstream areas [34,48].

The phenomenon of higher microplastic abundance in mangrove zones compared to adjacent unvegetated tidal flats has been widely documented [15,49]. However, variations in microplastic levels within mangrove systems require more detailed assessment. In this study, interior mangrove habitats showed significantly higher microplastic abundance (292.86 ± 31.49 items·kg^−1^) compared to edge zones (142.50 ± 17.87 items·kg^−1^). This aligns with the expected mechanisms: complex hydrodynamic pathways and dense root networks in interior habitats reduce flow velocity, promoting microplastic sedimentation [50]. However, boundary sediments contained higher proportions of films (potentially from floating debris) and larger particles, suggesting tidal sorting preferentially removes low-density, coarse microplastics. Based on field observations and previous research investigations, sediments in the mangrove edge are often covered with plastic woven bags, fishing nets, and ropes. This plastic debris is easily physically intercepted by the mangroves and trapped in the edge areas [51,52]. Once broken down, they may result in an increased proportion of PE and PA microplastics. As the overall abundance of microplastics decreases in the edge habitat, the abundance of other types of microplastics (such as PET) may decline. In contrast, boundary sediments contained higher proportions of films (potentially from floating debris) and larger particles, suggesting tidal sorting preferentially removes low-density, coarse microplastics. These findings highlight spatial heterogeneity in microplastic characteristics within mangroves, shaped by particle properties, hydrological conditions, and vegetative interception. These spatial patterns underscore the need for targeted sampling approaches in different mangrove zones. As shown in atmospheric microplastic studies, site-specific sampling methods are crucial for accurately capturing microplastic distributions [53]. Similarly, in mangrove systems, future research should employ adaptive sampling strategies that account for hydrological conditions and the distinct microplastic characteristics of interior and edge habitats. Additionally, the role of the seawall is worth noting. Although there is no direct evidence to prove that the seawall affects the distribution of microplastics, its influence on hydrodynamics and sedimentary conditions suggests that it may have contributed to the high abundance of microplastics in the mangrove interior habitat.

Beyond abundance and composition, analyzing microplastic diversity provides richer insights into their spatiotemporal distribution [27,54]. This study identified distinct spatial differences in microplastic diversity across two gradients. From upstream to downstream, both Shannon–Wiener and Simpson diversity indices showed decreasing trends, while Pielou’s evenness index was slightly higher at edge areas. This pattern likely reflects the complexity of pollution sources upstream (e.g., multiple terrestrial inputs) versus tidal homogenization downstream, where low-density polypropylene (PP) tends to migrate seaward under hydrodynamic forces [55,56]. In estuarine environments, hydrological conditions serve as the primary driver shaping ecological patterns. Enhanced hydrodynamic forces allow only larger, denser plastic particles to settle without being washed away [54]. Notably, mangrove interior habitats exhibited significantly higher Shannon–Wiener indices than boundaries, suggesting that despite the limited spatial scale of this study, interior zones act as “pollution sinks”, receiving inputs through multiple pathways (tidal transport, atmospheric deposition, and terrestrial runoff). The lower diversity at edges may result from tidal physical sorting that selectively retains particles with specific sizes and densities [55]. These findings are consistent with observations in Beibu Gulf mangroves, South China [57], further supporting mangrove interiors as microplastic diversity hotspots.

As biodiversity-rich ecosystems at estuarine freshwater-saltwater interfaces, mangroves face significant ecological risks from microplastic pollution. Comparative risk assessments revealed relatively lower risks in Zhangjiang mangroves versus other systems: Sanyahe mangroves, Hainan, China (PLI = 2.28 [level I], PHI = 58.38 [level III]) [32]; Laem Chabang mangroves, eastern Thailand (PLI = 2.19 [level I], PERI = 2259.6 [level V]) [58]; and Zhanjiang mangroves, South China (PLI = 2.1 [level I], PHI = 4.7 [level I], PERI = 222.9 [level II]) [59]. While current risk indices (PLI/PHI/PERI) focus on polymer hazard profiles and environmental loads, the bioavailability of microplastics—particularly PVC and PS fragments dominating our samples—warrants attention. These dense, hydrophobic polymers readily adsorb persistent organic pollutants and resist biodegradation, enhancing their bioaccumulation potential in benthic organisms like fiddler crabs and mangrove snails [60]. While no significant upstream-downstream risk gradient was detected, Site A showed slightly elevated risks due to the detection of high-hazard-score PVC polymers. The vertical trophic transfer of these particle-bound contaminants may pose cascading threats to fish and wading birds, though this requires verification through tissue analysis across food web tiers. Notably, ecological risks within the interior mangrove habitats were much higher than those in the boundary zones. This was primarily due to two key factors: the significantly greater abundance of microplastics in the interior habitats and the higher prevalence of high-risk polymers, such as PVC, PS, and PE, in the interior sediments. Future monitoring should integrate ecotoxicological assays (e.g., biomarker responses in sentinel species) with physical risk indices to better predict ecosystem-scale impacts.

Furthermore, this study uncovered distinct spatial variations in microplastic morphology and size within mangroves. However, current ecological risk assessment frameworks predominantly focus on abundance and polymer toxicity, rarely incorporating critical parameters like particle size and shape. For instance, particles < 500 μm can penetrate biological barriers and prolong gastrointestinal retention, intensifying toxic accumulation [61,62], while the dominant fragments and fibers (88.3%) may induce mechanical damage to digestive tracts or entanglement-related feeding inhibition [63,64]. Although morphology-toxicity linkage models have been proposed [65], mainstream methodologies still inadequately address morphological characteristics. We therefore advocate for developing multidimensional risk assessment systems that integrate shape and size parameters, enabling precise identification of microplastic risk hotspots in sensitive ecosystems like mangroves.

This study provides an initial assessment of microplastic pollution in the Zhangjiang Estuary mangroves. However, the findings are based on a single dry-season sampling event (December) and seven strategically selected sites; furthermore, the visual identification methodology using 200× magnification limits the detection of smaller microplastics, likely resulting in an underestimation of particle concentration and an incomplete representation of the size range present. To comprehensively understand the spatiotemporal dynamics of microplastics in mangrove ecosystems, future studies should integrate broader spatial-temporal monitoring across seasons and additional sites, employing complementary techniques such as μ-FTIR analysis of directly filtered samples on replicate filters to capture smaller particles, coupled with analyses of seasonal drivers such as river discharge and coastal anthropogenic pressures.

## 5. Conclusions

This study reveals spatial differences in microplastic pollution across the Zhangjiang Estuary mangroves, confirming their role as traps for both land- and ocean-derived microplastics. The decreasing microplastic abundance from upstream to downstream reflects proximity to human activities and tidal sorting effects. Interior mangrove habitats showed significantly higher microplastic levels and diversity compared to edge areas, likely due to root-induced particle trapping and weaker tidal flushing. While overall ecological risks here were lower than in other global mangroves, interior zones exhibited elevated risks linked to high-risk polymers (PVC, PS, PE) and dominance of small particles (<500 μm). The prevalence of fragments, fibers, and small sizes emphasizes the need to incorporate physical traits into risk assessments. These findings support prioritizing protection of interior mangrove zones and implementing comprehensive monitoring to reduce microplastic threats in these sensitive coastal ecosystems.

## Figures and Tables

**Table 1:** Relative pollution level and potential ecological risk of microplastics in sites of the Zhangjiang Estuary.

Risk Category	Site						
	A	B	C	D	E	F	G
PLI	2.38	2.58	2.16	2.12	1.91	1.87	1.26
Hazard level	I	I	I	I	I	I	I
PHI	468.76	16.77	16.28	12.95	14.00	16.00	15.50
Hazard level	IV	III	III	III	III	III	III
PERI	9.38	0.34	0.33	0.26	0.28	0.32	0.31
Hazard level	I	I	I	I	I	I	I

Note: Roman numerals in the table represent hazard levels. Data from replicate sampling points at each site (e.g., A1 and A2, B1 and B2) were integrated to produce a merged dataset for each location.

**Table 2:** Risk assessment of microplastics in different habitats of Zhangjiang Estuary.

Risk Category	Habitats	
	Interior	Edge
PLI	2.66	1.72
Hazard level	I	I
PHI	506.18	54.08
Hazard level	IV	III
PERI	10.12	1.08
Hazard level	I	I

Note: Roman numerals in the table represent hazard levels. Data from all interior sampling points (A1–G1) and all edge sampling points (A2–G2) were separately integrated to generate merged datasets for interior and edge habitats.

## Data Availability

The data presented in this study are available on request from the corresponding author.

## Supplementary Materials

The following supporting information can be downloaded at: https://www.mdpi.com/article/10.3390/toxics13060494/s1, Table S1: Hazard score of MPs polymer type; Table S2: Risk level criteria for relative pollution level and potential ecological risk.
