# Supplementary material for: Distribution, Diversity, and Ecological Risks of Microplastics in Mangrove Ecosystems of a Southeastern Chinese Estuary
Source: Toxics. 2025 Jun 12;13(6):494. doi: 10.3390/toxics13060494 (PMC12197792; doi:10.3390/toxics13060494)
Supplement: Supplementary file 1 [file toxics-13-00494-s001.zip › toxics-3647595-supplementary.pdf]

Table S1. Hazard score of MPs polymer type.

Polymer	Hazard level	Hazard score
PA (Polyamide)	III	4
PE (Polyethylene)	I	11
PES (Polyester)	II	4
PET (Polyethylene terephthalate)	II	4
PP (Polypropylene)	II	1
PS (Polystyrene)	0	30
PVC (Polyvinyl chloride)	V	10001
RA (Rayon)	0	0

Reference: Lithner et al., 2011

Table S2. Risk level criteria for relative pollution level and potential ecological risk.

Risk category (Level)	Low (I)	Moderate (II)	Considerable (III)	High (IV)	Very high (V)
Pollution load index (PLI)	0–10	10–20	-	20–30	>30
Polymer hazard index (PHI)	0–1	1–10	10-100	100–1000	>1000
Potential ecological risk index (PERI)	0–150	150–300	300–600	-	>600

Reference: Vibhatabandhu et al., 2025; Shekoohiyan et al., 2022
